# Shifting the paradigm in the management of early prostate cancer

**DOI:** 10.1038/s41416-024-02641-7

**Published:** 2024-03-06

**Authors:** Vincent Gnanapragasam

**Affiliations:** 1 Cambridge Urology Translational Research and Clinical Trials Office, Cambridge, UK; 2 Division of Urology, Department of Surgery, University of Cambridge, Cambridge, UK

**Keywords:** Prostate cancer, Education

## Abstract

Outcomes from active surveillance have clearly shown that it is the optimal method of managing many early prostate cancers. Yet, clinician training and healthcare systems are still primarily focused on the “need to treat”. This comment explores the challenges and resource issues in future implementation of high-quality surveillance programmes.

## Commentary

Clinicians implicitly understand the definitions of locally advanced or metastatic prostate cancer, however the nomenclature for earlier disease states is not as well defined and differ depending on the country and guideline used. This is becoming increasingly important as many men will now present with prostate cancer that could be classified as “early”. The 25-year-old terminology of “risk” (based on biochemical relapse after surgery or radiotherapy) is no longer relevant or suitable in the context of modern disease management [1].

Perhaps one unifying theme across guidelines is that early cancers are those considered suitable for active surveillance (AS) as either a preferred or an equal option for management.

AS is a programme of monitoring prostate cancer that is considered to have a very low chance of metastasis and/or causing death. Modern AS involves a schedule of regular blood tests to monitor the Prostate Specific Antigen levels (PSA), interval MRI scans of the prostate and repeat prostate biopsies if indicated. The aim is to avoid over-treatment of cancers that are unlikely to cause harm in a patient’s natural lifetime. In the UK, NICE have defined cancer suitable for AS as those classified as NICE Cambridge Prognostic Groups (CPG) 1 and 2 (https://www.nice.org.uk/guidance/ng131) (*outside the UK the terms “low-risk” and “favourable-intermediate risk” continue to be used and correspond approximately to CPG1 and CPG2*). It is estimated that up to 1 in 3 men are diagnosed with CPG1/2 prostate cancer and potentially suitable for AS every year in England alone (approx. 20-25,000 men/year) [2].

Understanding of the long lead times of early cancer trajectories is now clearer with the reporting of seminal long-term observation and randomised control studies [3, 4]. These have shown equivalence of immediate intervention versus surveiilance in survival rates but continue to generate strong views for and against treatment; usually depending on the skillset of the proponent. This long natural history has arguably also helped underpin the genesis of “lesion only” ablative therapies which have so far shown better functional outcomes but not improved survival outcomes. The same long natural history mandates the more critical question of whether early cancers need any treatment at all at the time of diagnosis.

In the past decades AS has moved from the fringes of prostate cancer management into primetime; becoming in its own right an expanding and increasingly complex area of clinical management. Outcomes from active surveillance have clearly shown that it is the optimal method of managing CPG1 prostate cancer and there is strong evidence that CPG2 disease can equally be well managed with surveillance, although with more careful protocols. Where once research and discovery focused on surgical, radiotherapy or other intervention outcomes, AS now has its own rapidly growing literature on biomarkers, imaging, clinical protocols, criteria, intervention trials and artificial intelligence technologies. Indeed, the management of early prostate cancer represents the quintessential example of the need for personalised medicine i.e. tailored individualised risk-stratified management to avoid both over or under-treatment as well as over or under-monitoring. Key to this is the oft-neglected aspect of a holistic patient centric and not disease centric approach. Given that the peak age of diagnosis is in men over 70y and that male life expectancy (UK, ONS) is about 79 y, many men with early prostate cancers are unlikely to live long enough to see a survival benefit from treating their disease. National Cancer targets pay scant attention to this aspect, focused as they are on speed, rapid flow and definitive treatment timescale as the measurable outcome [5].

Individual contextualised counselling of prostate cancer is now more than ever imperative when discussing the value of treatment versus competing cause mortality. Research has shown that clinicians often over-estimate disease lethality and underestimate competing mortality effects [6]. Reasons cited for not using risk calculation tools and decision aids (even when free and web-based) include too many patients, lack of time, or practice inertia. This latter aspect is likely rooted in how urologists and oncologists are trained – i.e to be treatment rather than disease orientated specialists. As such clinicians are usually more comfortable replacing one treatment with another rather than with a no treatment approach. This also extends into AS programmes, with many studies showing high attrition rates from surveillance of what should be early indolent cancers. A mindset which views AS as a “waiting” option for men who will be eventually treated is different from one which views AS as the primary management option itself. Given these factors it is not surprising that there remain huge variations in over-treatment rates and AS conversion rates which have been shown time and time again in many health care systems [7, 8].

Evidence is growing that well-constructed risk-based surveillance protocols, can streamline follow-up and have low attrition rates [9] (Table 1). However, this needs a dedicated team knowledgeable in early prostate cancer as well as resources. It cannot continue to be simply managed in any available urology or oncology clinical and general capacity. But is urological practice in the UK (or elsewhere) ready to cope with the adoption of such structured AS as a major management option? Unlike any interventional treatment, AS lacks any training requirements, professional oversight, quality standard, national audit or dedicated resources. Lack of resourcing may also come into play in an unconscious bias to guide patients towards treatment which has a much more defined pathway. After all, hospital clinic and appointment capacity are usually built around the “treat and discharge” model. If AS instead were to be given the same attention and priority as treatment interventions it can be expected that clinician engagement would also increase.

**Table 1:** Examplar of a risk-based Active surveillance programme for early prostate cancer.

STRATCANS group	Inclusion criteria	Follow up schedule
**1**	Cambridge Prognostic Group 1 AND PSAd<0.15	3 monthly PSA 18 monthly out-patients telephone MRI Likert 1-2 - repeat at 3 years MRI Likert 3 - repeat at 18 months MRI Likert 4-5 – repeat at 12 months No routine re-biopsy Triggered re-biopsy if any change
**2**	Cambridge Prognostic Group 2 OR PSAd ≥ 0.15	3 monthly PSA 12 monthly out-patients telephone MRI Likert 1-2 - repeat at 3 years MRI Likert 3 - repeat at 18 months MRI Likert 4-5 – repeat at 12 months Re-biopsy at 3 years* Triggered re-biopsies if any change
**3**	Cambridge Prognostic Group 2 AND PSAd ≥ 0.15	3 monthly PSA 6 monthly out-patients telephone MRI (any Likert)- repeat at 12 months Re-biopsy at 3 years* Triggered re-biopsies if any change

STRATified CANcer Surveillance (STRATCANS) [9] is an example of a risk based AS monitoring programme. It is based on different progression risks from the diagnostic clinic-pathological and imaging features. It defines a rational evidence base approach for out-patient appointments, PSA testing, MRI scans and recommendations for biopsy (PSAd- PSA density). Further information can be found at https://www.stratcans.com. (Likert- refers to the 5-point MRI scale,* Option to omit and discuss with patient).

Another integral need is training and educating future clinicians in a better understanding of early prostate cancer and the nuances of the disease rather than just as something to treat. The primary imperative of research into any disease is to understand its biology, natural history and to develop new targeted interventions. Taking this to its natural conclusion, if early detection and mechanistic research is successful, it is conceivable there will come a time when early cancer may be manageable with drug/medical therapy alone. The need for and use of ablative or removal therapies may become limited and instead we will need disease specialists who can provide holistic and multi-aspect management.

When we consider how any sub-speciality practice emerged, it usually starts when new data and evidence recognises a unique clinical need and expert knowledge base. This coupled with a growing patient demographic argues strongly for a change in how we resource early cancer management and train new clinicians to meet this emerging landscape. In this way we may be able to provide dedicated disease specialists for this growing demographic of future patients for whom prostate cancer may just be one part of their overall medical co-morbidity.
